# Evaluation of efforts in untrained Wistar rats following exercise on forced running wheel at maximal lactate steady state

**DOI:** 10.20463/jenb.2017.0040

**Published:** 2017-03-31

**Authors:** Sajjad Rezaei, Hamid Agha-alinejad, Mahdieh Molanouri Shamsi, Mahvash Jafari, Fabricio Azevedo Voltarelli, Alireza Naderi, Conrad Earnest

**Affiliations:** 1 Physical Education & Sport Sciences Department, Faculty of Humanities, Tarbiat Modares University, Tehran Iran; 2 Department of Biochemistry, Faculty of Medicine, Baqiyatallah University of Medical Sciences, Tehran Iran; 3 Graduation Program of Physical Education, Faculty of Physical Education, Federal University of Mato Grosso, Cuiabá Brazil; 4 Department of Sport Physiology, Boroujerd Branch, Islamic Azad University, Boroujerd Iran; 5 Exercise Science and Sport Nutrition, College Station, Texas A&M University, Texas USA

**Keywords:** Exercise intensity, Forced running wheel, Lactate threshold, Maximal lactate steady state, Rat

## Abstract

**[Purpose]:**

We aimed to examine the effect of running speed on metabolic responses associated with maximal lactate steady state (MLSS) in rats during forced running wheel (FRW) exercise.

**[Methods]:**

Forty male adult Wistar rats were divided into seven groups. The blood lactate threshold and peak running speed were determined for an incremental power test group. Five groups participated in constant power tests at intensities 10, 13, 14.5, 16, and 17.5 m/min to determine MLSS and a non-exercise group was chosen as the control. Animals were euthanized immediately after constant power tests and their corticosterone, non-esterified fatty acid (NEFA), blood glucose, and creatine kinase (CK) levels analyzed. The differences among groups were identified by one-way analysis of variance (p < 0.05).

**[Results]:**

Blood lactate threshold corresponded a running intensity of 15 m/min, while MLSS was determined to be 16 m/min. Serum corticosterone concentrations were significantly higher in 14.5, 16, and 17.5 m/min groups (298.8±62, 338.3±65, and 354±26 nM, respectively) as compared to that in the control group (210.6±16 nM). Concentrations of NEFA observed in groups 13, 14.5, 16, and 17.5 m/min (662.8±24, 702.35±69, 718.4±34, and 752.8±77 μM, respectively) were significantly higher than those in 10 m/min and control groups (511.1±53 and 412.1±56 μM, respectively). The serum CK concentration recorded for group 17.5 m/min (372.4±56 U/L) was higher than those recorded for other groups.

**[Conclusion]:**

The speed above 16 m/min on FRW resulted in increased physiological demands and muscle damage in untrained healthy Wistar rats.

## INTRODUCTION

Rats are typically used to simulate human physical conditions associated with exercise. However, the direct application of animal study results to humans requires an exact methodology. Exercise in rats is generally “forced,” wherein several means are employed to keep the animals moving. Translational applications from rat studies necessitate adequate control over the type, intensity, and duration of exercise. 

Evidences suggest intrinsic differences between the forced and voluntary exercise models^1^. For example, forced exercise exacerbates a stress response as compared to voluntary exercise^2^. Thus, it may be difficult to establish sufficient parallel findings in humans using these models and presents a need for a more precise model that can translate and extend findings in animal models to humans. 

Aerobic fitness is typically determined by maximal oxygen uptake (VO_2max_). As the equipment used for the estimation of VO_2max_ in rats is expensive, alternative methods are followed for determination of the effort intensity. Anaerobic threshold, or the aerobic-anaerobic transition phase, is a widely applied method for the determination of the extent of an animal’s effort during physical exercise^3-6^. The investigation of changes in the blood lactate concentration during incremental power test (INCP) and the use of gold standard protocol of maximal lactate steady state (MLSS) are precise methods for the identification of anaerobic threshold^7^ and, as a result, the intensity of the animal’s effort during physical exercise^4, 5^. 

Changes in parameters other than blood lactate concentration are observed as a response to the animal’s effort to maintain homeostasis during physical exercise. Depending upon the exercise type and intensity, the concentration of stress hormones such as corticosterone— a measure of the hypothalamic-pituitary-adrenal (HPA) axis activity— increases following physical exercise^8 ,10^. The increase in corticosterone level in response to exercise induced stress is attributed to its capacity to supply energy. Further, the analysis of blood glucose, serum free fatty acid, blood lactate, and serum corticosterone concentrations immediately after physical exercise seems to be highly useful in the assessment of exercise-induced stress^11^. 

Forced running wheel (FRW) enables researchers to determine the intensity and duration of exercise without using a stimulator. Given its complex movement patterns, the physical exercise in this ergometer offers many benefits for studying the memory of healthy and unhealthy rat models^1, 12, 13^. However, FRW exhibits limitations in terms of the application of the desired intensity^1, 14, 15^. The primary aim of this study was to examine blood lactate levels and biochemical markers of stress following INCP and MLSS protocols. We hypothesize that changes in stress biomarkers at intensities above lactate threshold (LT) are higher than those at intensities below and around LT. 

## METHODS

### Animals

All experiments involving animals were conducted according to the policies of the Iranian Convention for the Protection of Vertebrate Animals Used for Experimental and Other Scientific Purposes, and the protocol was approved by the Ethics Committee of the School of Medical Sciences, Tarbiat Modares University (TMU), Tehran, Iran. Adult (8-week old) male Wistar rats (N = 40) were obtained from the Pasteur Institute of Iran. Animals weighing 200–240 g at the start and 250–300 g at the end of the experiment were housed in standard cages (five rats per cage) at 22–24°C and 50%–60% humidity with a 12:12 h light dark cycle. Feed and water were provided *ad libitum*. 

### Selection of runner rats and familiarization with FRW

The selection of runner rats was based on 10 consecutive days of running at 10 m/min for 5 min. Rats that showed successful performance for at least 9 out of 10 days were considered as the runner rats^16^. These rats were adapted to the FRW (Lafayette Instruments, Lafayette, IN, USA) for 3 weeks^8^. 

### Procedures and experimental groups

The runner rats were assigned to seven experimental groups (n = 5–6) characterized by the type and intensity of exercise. The LT and peak running speed for the INCP test was determined for one experimental group, followed by the participation of other groups in a series of constant power (CP) tests at speeds 10, 13, 14.5, 16, and 17.5 m/min (above and below LT). A control group was used to identify the level of stress at basal condition. CP test groups were sacrificed immediately after the test to examine stress indicators. 

### Determination of LT and INCP

An incremental power test was performed to determine LT and peak speed. The test began at the speed of 2.5 m/min, with an increase of 2.5 m/min at the end of every 3-min stage. Blood samples were collected between each stage, while rats continued running until exhaustion. The speed at which rats changed their movement pattern (five times in 3 min), repeatedly grabbing the wheel and refraining from running, was regarded as the exhaustion speed^14, 17^. We determined LT by visual inspection of the plotted graphs between lactate concentration and speed. A second order polynomial regression equation was used to examine the increase of ≥ 1 mM in lactate concentration^18^. 

### Determination of MLSS

We carried out several CP tests at 10, 13, 14.5, 16, and 17.5 m/min to determine MLSS using speeds set between 40%–70% of the peak speed derived from the INCP group. Animals performed these tests after a 10-min warm-up at 2.5 m/min, and the tests lasted for 25 min of continuous exercise or until exhaustion. A 30-s interval was included after every 5 min for blood collection. The MLSS was considered as the highest intensity of the CP test with less than 1 mM increase in blood lactate concentration between 10^th^ and 25^th^ min^8^. The MLSS concentration was calculated as the mean blood lactate concentration measured at 10, 15, 20, and 25 min of test. 

### Blood sample collection for lactate analysis

Blood samples were collected through a small incision in the distal tail portion^19^. About one drop of blood was taken at the start and at 5-min intervals of CP tests, and before and after every 3-min stage in INCP test. Blood lactate concentration was determined by the photometric method, using a Lactate Scout analyzer (SensLab GmbH, Germany)^20^. 

### Euthanasia and biochemical analysis

Rats were anesthetized with a mixture of ketamine (30–50 mg/kg body weightBW, IP) and xylazine (3–5 mg/kg BW, IP) immediately after the completion of each test. Blood was collected into dry tubes by cardiac puncture after thoracotomy. Coagulated whole blood was centrifuged at 4,000 ×g for 10 min and the serum stored at -80°C in microcentrifuge tubes until analysis. Whole blood was collected from the tail vein to measure glucose levels after exercise and before anesthesia using a glucometer (Accu-ChEK, Germany). Serum corticosterone level was assessed using corticosterone RIA kit for rat (DRG, EIA-4164, Germany). Non-esterified fatty acid (NEFA) and serum creatine kinase (CK) levels were determined using commercial kits (Zell Bio, Germany). All assays were carried out according to the manufacturers’ instructions. 

### Statistical analyses

All analyzed data were normally distributed, as assessed by the Kolmogorov-Smirnov test (p > 0.05). Differences between groups were compared by one-way analysis of variance (ANOVA) followed by Tukey posthoc test. The value of p < 0.05 was considered statistically significant. SPSS version 16.0® and Microsoft Excel® were used and data presented as mean ± standard deviation (SD) unless otherwise noted. 

## RESULTS

### Blood lactate analysis

**Figure 1.**
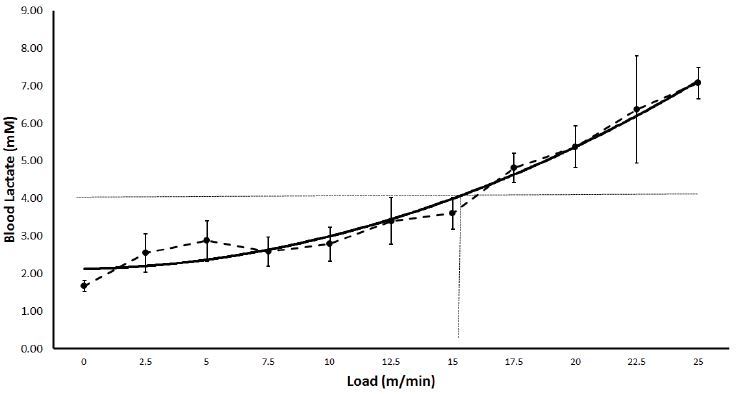
Visual identification of lactate threshold in rats while running on FRW during the incremental power test. Each point shows blood lactate (mM) after 3 min of exercise with incremental load.

Figure 1 shows changes in blood lactate concentration in the group subjected to the INCP test. The intensity corresponding to LT (3.6 ± 0.45 mM) was determined to be 15 m/min, whereas the average peak speed recorded was 25 m/min. 

**Figure 2.**
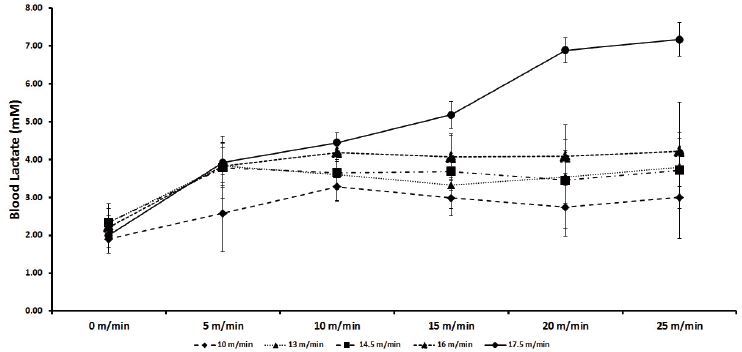
Mean blood lactate concentration during constant power tests to determine MLSS in Wistar rats. Animals (n = 5–6) participated in five tests at different speeds.

**Figure 3.**
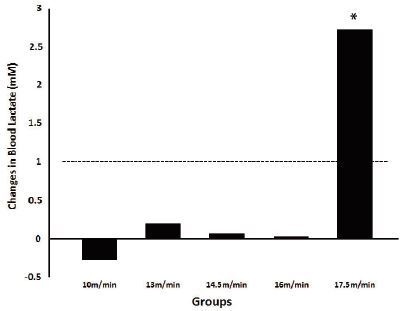
Changes in blood lactate concentration between 10 and 25 min during constant power tests at different intensities of exercise on FRW. *p < 0.05 in comparison with groups 10, 13, 14.5, and 16 m/min.

The blood lactate concentration recorded at speeds 10, 13, 14.5, 16, and 17.5 m/min during the CP tests is shown in Figure 2. An increase in the blood lactate concentration was observed for 17.5 m/min group as compared to that in other groups. Some animals had reached exhaustion before the test completion. Changes below 1 mM in blood lactate concentration were reported for other groups between 10 and 25 min (Figures 2 and 3, p < 0.05). The highest speed of running on FRW with < 1 mM change in blood lactate concentration between 10 and 25 min was 16 m/min; thus, 16 m/min was regarded as the intensity equivalent to MLSS workload (intensity) and was around 65% of the peak speed recorded in the INCP test. The lactate concentration reported at MLSS intensity was 4 ± 0.1 mM. 

### Stress biomarkers

**Figure 4.**
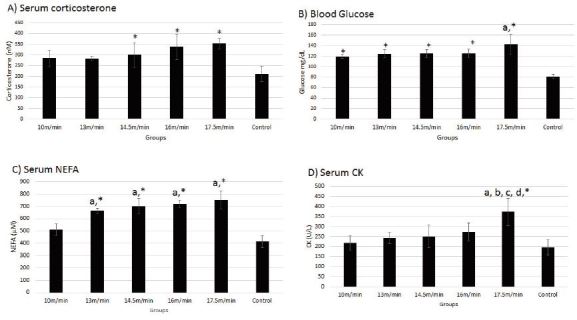
Concentrations (mean ± SD) of serum corticosterone (A), blood glucose (B), serum NEFA (C), and serum creatine kinase (D) immediately after exercise at different intensities based on MLSS protocol. *p < 0.05 in comparison with the control group; a in comparison with group 10 m/min; b in comparison with group 13 m/min; c in comparison with group 14.5 m/min; d in comparison with group 16 m/min.

Our results showed an increase in levels of corticosterone hormone, NEFA, glucose, and CK immediately after exercise on FRW (Figure 4). A significant increase in the concentration of corticosterone was observed in groups 14.5, 16, and 17.5 m/min (298.8 ± 56.9, 338.3 ± 6, and 354 ± 24.2 nM, respectively) as compared with that in the control group (210.6 ± 35.7, p < 0.05, Figure 4A). A significant difference was observed in blood glucose level between all groups and the control (81.2 ± 3.9 mg/dL). Moreover, the concentration of glucose in group 17.5 m/min (142.8 ± 19.21 mg/dL) was significantly higher than that reported in group 10 m/min (118.8 ± 3.48 mg/dL) (p < 0.05, Figure 4B). Concentrations of NEFA observed in groups 13, 14.5, 16, and 17.5 m/min (662.8 ± 22, 702.35 ± 63, 718.43 ± 31, and 752.86 ± 71 μM, respectively) were significantly higher than those in 10 m/min and control groups (511.1 ± 47 and 412.12 ± 52 μM, respectively). No significant difference in NEFA concentration was observed between the control and 10 m/min groups (p < 0.05, Figure 4C). The serum CK concentration recorded for group 17.5 m/min (372.4 ± 68 U/L) was higher than the concentrations recorded for other groups (p < 0.05, Figure 4D). In other groups, the serum CK concentration increased with increased intensity of exercise, but showed no statistical significance. 

## DISCUSSION

Our study showed a correlation between exercise intensity and MLSS in Wistar rats running on FRW. These findings are important for examining exercise intensity and performance in murine studies because it allows researchers to identify the intensity of physical activities associated with MLSS^21^, which is regarded as a standard method for the analysis of aerobic capacity as well as for the detection of highest aerobic power^7^. In this study, MLS intensity was determined to be 16 m/min on FRW (Figure 2), according to a protocol similar to that established by Contarteze et al^8^. 

The MLSS intensity determined in our study corresponded to the blood lactate concentration of 4.1 ± 0.4 mM. Manchado et al. reported similar values (3.9 ± 0.3 mM at the speed of 20 m/min) in rats running on a treadmill^16^. Given the sensitivity of lactate production in the muscles used during exercise and the movement pattern, the observed difference in the intensity of MLSS can be related to the ergometer used in studies^22^. On treadmill, the movement is directed forward on a flat and linear path, whereas running on FRW necessitates the animal to grab the bars. Thus, it can be argued that the complexity of the wheel impels the animal to put more efforts for maintaining its speed of running. Furthermore, the INCP test results indicated a sudden rise (> 1 mM) in blood lactate concentration at 15 m/min. Hence, the concentration equivalent to LT was visually determined to be 3.6 ± 0.45 mM at this speed. 

Pilis et al.^5^ reported an LT value of 4 mM at 25 m/min in rats running at increasing speed on the treadmill. Thus, the type of the exercise (treadmill versus FRW) as well as the protocol may influence LT. In Wistar rat, LT identified by an INCP test is usually similar to its MLSS^23^. This is contradictory to results observed in our study. The absence of any previous knowledge of FRW parameters such as the speed at, and duration of, each stage in the INCP test may have affected the LT value. 

Ferreira et al. found a correlation between exercise intensity and MLSS and showed that MLSS occurred at 60% of maximal speed in the INCP test^24^. Our result showed that the intensity of MLSS on FRW correlated to 65% of the peak speed obtained from the INCP test. Given the dependence of MLSS intensity on aerobic capacity and the sensitivity of aerobic exercises^4^, this value can be used to design training programmes of aerobic exercises and analyze endurance performance of healthy rats running on forced wheel. 

The elevated secretion of adrenocorticotropic hormone in response to stress (especially when the need for vital substrates increases) results in the rise in the activity of HPA axis, thereby increasing the concentration of glucocorticoids such as corticosterone^25^. Glucocorticoids break down amino acids and fats from storage sites to release glucose as an energy source for other tissues^11^. Thus, the increase in blood glucose, NEFA, and lactate levels during exercise can be attributed to the rise in corticosterone level. We observed a significant increase in the concentration of blood glucose in all exercise groups as compared with that in the control (resting) group. The increase in glucocorticoids was associated with 6–10 times increase in liver glycogenolysis and reduction in the rate of cellular glucose consumption, thereby increasing the blood glucose levels^26, 27^. Therefore, the increased rate of gluconeogenesis and reduction in cellular glucose consumption at exercise intensities higher than MLSS may increase the blood glucose concentration. Although no significant difference was observed between the corticosterone concentrations in 10 m/min and control groups, it was surprising that the blood glucose concentration reported for the 17.5 m/min group (intensity above MLSS) was significantly higher than that in the 10 m/min group. Glucocorticoids also stimulate the release of free fatty acids either directly or indirectly via the parasympathetic system^28^. The NEFA concentration reported in our study was significantly higher in all groups as compared to 10 m/min and control groups. 

Although we have studied only the stress-related biomarkers in response to acute exercise in rats, it should be noted that stress induction with high-intensity exercise may elicit muscle damage. The increase in the CK activity in healthy subjects following high-intensity or prolonged exercise is an indicator of exercise-induced muscle damage^29^. We observed an increase in the CK activity in 17.5 m/min group as compared to that in other groups (Figure 4D). The CK activity fails to reflect the amount of muscle mass involved in exercise. However, alterations in the CK activity was observed with the accumulation of blood lactate, glucose, NEFA, and corticosterone, indicative of the increased performance pressure on muscles during high-intensity exercise. It therefore seems that a little change in the exercise intensity around MLSS (16–17.5 m/min) may significantly affect the physiological response to exercise on FRW. Further studies for the determination of precise intensity should be carried out at 16 m/min or below at the start of the aerobic training program or above 16 m/min for interval exercise in healthy untrained rats using FRW. 

A well-designed exercise protocol is of paramount importance in exercise studies. We performed a direct measurement of the exertion in animals during exercise. Researchers have traditionally applied low-to-moderate or high-intensity interval protocols, owing to the uncertainty regarding the exertion of rats using FRW^1, 14, 15^. While studies have shown that rats are reluctant to run at speed ≥ 20 m/min on FRW, literature suggests anaerobic threshold in rats running on treadmill at around 20 m/min^5, 16^. Researchers have incorporated changes in the FRW platform to encourage animals to run for 1 h at ≥ 20 m/min^30, 31^. However, any change in the movement pattern alters the sport-specific mass of working muscle^21^ and may affect both the perceived exertion and exercise intensity. Given some of animals in our study were unable to complete the CP test at 17.5 m/min, indicating that untrained rats require extra effort at speed above LT. Therefore, it could be argued that the animal’s reluctance to run at such high velocities for a long time is related to the exercise-induced fatigue, not the ergometer deficiency. 

Our study provides a measure of intensity of exercise at a given running speed on FRW. The exercise at different intensities based on aerobic-anaerobic transition phase induced endocrine metabolic stress responses (measured as an increase in corticosterone concentration) in animals. The speed above of 16 m/min on FRW resulted in increased physiological demands and muscle damage in untrained healthy Wistar rats. We suggest that researchers use an initial speed of ≤16 m/min for aerobic training or >16 m/min during interval training of untrained rat, using FRW, to achieve expected adaptations to exercise. 
